# Tyrosine Phosphatase PTPRO Deficiency in ERBB2-Positive Breast Cancer Contributes to Poor Prognosis and Lapatinib Resistance

**DOI:** 10.3389/fphar.2022.838171

**Published:** 2022-04-01

**Authors:** Hongmei Dong, Liang Du, Songwang Cai, Wan Lin, Chaoying Chen, Matthew Still, Zhimeng Yao, Robert P. Coppes, Yunlong Pan, Dianzheng Zhang, Shegan Gao, Hao Zhang

**Affiliations:** ^1^ Institute of Precision Cancer Medicine and Pathology, School of Medicine, Department of General Surgery, The First Affiliated Hospital of Jinan University, Minister of Education Key Laboratory of Tumor Molecular Biology, Jinan University, Guangzhou, China; ^2^ Departments of Biomedical Sciences of Cells and Systems, Section Molecular Cell Biology and Radiation Oncology, University Medical Center Groningen, University of Groningen, Groningen, Netherlands; ^3^ Graduate School, Shantou University Medical College, Shantou, China; ^4^ Department of Thoracic Surgery, The First Affiliated Hospital of Jinan University, Guangzhou, China; ^5^ Cancer Research Center, Shantou University Medical College, Shantou, China; ^6^ Department of Clinical Laboratory, The First Affiliated Hospital of Hunan Traditional Chinese Medical College (Hunan Province Directly Affiliated TCM Hospital), Zhuzhou, China; ^7^ Department of Bio-Medical Sciences, Philadelphia College of Osteopathic Medicine, Philadelphia, PA, United States; ^8^ Department of General Surgery, The First Affiliated Hospital of Jinan University, Jinan University, Guangzhou, China; ^9^ Henan Key Laboratory of Microbiome and Esophageal Cancer Prevention and Treatment, Henan Key Laboratory of Cancer Epigenetics, Cancer Hospital, The First Affiliated Hospital (College of Clinical Medicine) of Henan University of Science and Technology, Luoyang, China; ^10^ Department of General Surgery, The First Affiliated Hospital of Jinan University, Institute of Precision Cancer Medicine and Pathology, School of Medicine, Minister of Education Key Laboratory of Tumor Molecular Biology, Jinan University, Guangzhou, China

**Keywords:** PTPRO, phosphatase, tyrosine kinase, ERBB2-positive breast cancer, prognosis, lapatinib resistance

## Abstract

Despite the initial benefit from treating ERBB2-positive breast cancer with tyrosine kinase inhibitor lapatinib, resistance develops inevitably. Since the expression of protein tyrosine phosphatase receptor-type O (PTPRO), a member of the R3 subfamily of receptor protein tyrosine phosphatases (PTPs), is inversely correlated with the aggressiveness of multiple malignancies, we decided to explore the correlation between PTPRO and lapatinib resistance in ERBB2-positive breast cancer. Results of immunohistochemical (IHC) staining and the correlation analysis between the expression levels of PTPRO and the clinicopathological parameters indicate that PTPRO is downregulated in cancer tissues as compared with normal tissues and negatively associated with differentiation, tumor size, tumor depth, as well as the expression of ERBB2 and Ki67. Results from Kaplan–Meier analyses indicate that lower expression of *PTPRO* is correlated with shorter relapse-free survival for patients with ERBB2-positive breast cancer, and multivariable Cox regression analysis found that PTPRO can potentially serve as an independent prognostic indicator for ERBB2-positive breast cancer. Results from both human breast cancer cells with PTPRO knockdown or overexpression and mouse embryonic fibroblasts (MEFs) which derived from *Ptpro* ^*+/+*^ and *Ptpro* ^*−/−*^ mice with then stably transfected plasmid FUGW-Erbb2 consistently demonstrated the essentiality of PTPRO in the lapatinib-mediated anticancer process. Our findings suggest that PTPRO is not only able to serve as an independent prognostic indicator, but upregulating PTPRO can also reverse the lapatinib resistance of ERBB2-positive breast cancer.

## Introduction

Breast cancer is by far the most common cancer and the leading cause of cancer-related mortality in women worldwide and therefore breast cancer presents a significant burden for public health (DeSantis et al., 2017; Bray et al., 2018). ERBB2, a member of the ERBB family of receptor tyrosine kinases (RTKs) (EGFR/ERBB1, ERBB2, ERBB3, and ERBB4), is overexpressed in approximately 15–20% of human breast cancers and its expression is highly associated with poorer overall survival (Nahta and Esteva, 2006; Murthy et al., 2016; Ethier et al., 2021). ERBB2 status is a determinant for stratifying invasive breast cancer concerning ERBB2-directed targeted therapy (Ahmed et al., 2015; Goutsouliak et al., 2020; Oh and Bang, 2020), including ERBB2-targeted monoclonal antibodies and small-molecule tyrosine kinase inhibitors such as lapatinib (Xia et al., 2005). Unfortunately, only about 24% of patients respond to lapatinib treatment and most of the responders become lapatinib resistant (D'Amato et al., 2015). Thus, understanding the molecular mechanisms of lapatinib resistance and developing new therapeutic strategies are urgently needed.

Protein tyrosine phosphatase receptor-type O (PTPRO), a member of the R3 subfamily of receptor protein tyrosine phosphatases (PTPs), has been found to correlate negatively with clinical aggressiveness in different malignancies (Motiwala et al., 2003; Motiwala et al., 2004; You et al., 2012; Hou et al., 2013; Huang et al., 2013; Dong et al., 2017a; Gan and Zhang, 2020). We and others have demonstrated that PTPRO is capable of dephosphorylating RTKs such as ERBB2 and EPH receptors (Shintani et al., 2006; Dong et al., 2017a) and repressing tumor growth (Motiwala et al., 2003; Motiwala et al., 2004; Dong et al., 2017a). In this study, we explored the correlation between PTPRO and lapatinib resistance in ERBB2-positive breast cancer. We found that 1) reduced levels of PTPRO correlate with poorer clinical outcomes; and 2) overexpressed PTPRO in lapatinib resistant ERBB2-positive breast cancer cells is capable of reversing lapatinib-resistance.

## Materials and Methods

### Patients and Tissue Samples

Surgically treated female breast cancer patients (*n* = 180) with confirmed pathology of invasive ductal carcinoma were included, and their clinicopathological characteristics are summarized in Table 1. Breast cancer tissues were obtained from the patients when undergoing surgical treatment at the Department of Surgery, Cancer Hospital affiliated to Shantou University Medical College, during the period from 2010 to 2013. All patients received primary treatment by surgery followed by adjuvant radiotherapy, chemotherapy, or hormone therapy. The mean age of the patients was 50 years (range = 19–78 years). The histological types and grades of the primary tumors were determined according to a system modified from the WHO classification. Tumor staging was defined according to the 7th Edition of the American Joint Committee on Cancer TNM Staging manual. For all cases, representative parts of the primary invasive ductal carcinoma, along with adjacent noncancerous tissue were frozen in liquid nitrogen-cooled isopentane at the time of diagnosis and stored at a temperature below -80°C until use. The rest of the tissue was fixed in buffered formalin, embedded in paraffin, and used for routine histology. Clinical research protocols of this study were reviewed and approved by the Ethics Committee of Shantou University Medical College (IRB serial number: # 04–070). Written informed consents were obtained from patients in accordance with principles expressed in the Declaration of Helsinki.

**TABLE 1:** Relationship between PTPRO expression and clinicopathologic variables in tissue samples of breast cancer patients (*n* = 180).

Variables	No. of samples	PTPRO expression		*P*
		Negative, no. (%)	Positive, no. (%)	
All samples	180	111 (61.7)	69 (38.3)	—
Age (years)				
≤50	105	64 (61.0)	41 (39.0)	0.816
>50	75	47 (62.7)	28 (37.3)	
Histologic differentiation				
Well	26	10 (38.5)	16 (61.5)	0.031
Moderate	101	66 (65.3)	35 (34.7)	
Poor	53	35 (66.0)	18 (34.0)	
Tumor size				
≤2	31	13 (41.9)	18 (58.1)	0.013
>2	149	98 (65.8)	51 (34.2)	
Tumor depth				
T_1_/T_2_	138	77 (55.8)	61 (44.2)	0.003
T_3_/T_4_	42	34 (81.0)	8 (19.0)	
Lymph node metastasis				
N0	108	70 (64.8)	38 (35.2)	0.287
N1	72	41 (56.9)	31 (43.1)	
Stage				
I/II	124	72 (58.1)	52 (41.9)	0.139
III/IV	56	39 (69.6)	17 (30.4)	
ERBB2 expression				
Negative	115	64 (55.7)	51 (44.3)	0.027
Positive	65	47 (72.3)	18 (27.7)	
Ki67 expression				
<20	38	3 (7.89)	35 (92.1)	0.000
≥20	142	108 (76.1)	34 (23.9)	

### Immunohistochemical Analysis

Four-μm-thick sections from representative tumor areas and normal mammary gland tissue from surgical specimen tissue blocks were processed according to standard immunohistochemistry (IHC) protocols (Dong et al., 2017b; Wang et al., 2019; Wang et al., 2020; Wang et al., 2021a) and stained with antibodies against PTPRO (Cat. No. HPA034525, Sigma-Aldrich, St. Louis, United States), and Ki67 (Cat. No. #9449, Cell Signaling Technology, Danvers, MA, United States). Sections incubated with immunoglobulin (Ig)G of appropriate species (rabbit or mouse) as the primary antibody were used as negative controls.

The percentage of positively stained cells was scored using the following scales: 0, no staining of cells in any field; 1, ≤10%; 2, 11–50%; 3, 51–75%; 4, >75%. The intensity of staining was scored using the following scales: 1+, weak staining; 2+, moderate staining; 3+, strong staining. Percentage (P) and intensity (I) of nuclear or cytoplasm or membrane expression were multiplied to generate a numerical score (S = P*I). The immunoreactivity was evaluated by two different pathologists with no prior knowledge of patient data. When the opinions of the two evaluators were different, the agreement was then compared and discussed.

### Analysis of Breast Cancer Microarray Datasets

Raw data from 1,079 breast cancer gene expression profiles from 5 independent datasets (GSE3494, GSE7390, GSE6532, GSE1456, and GSE2034) were collected from NCBI Gene Expression Omnibus (GEO) (Pawitan et al., 2005; Wang et al., 2005; Sotiriou et al., 2006; Desmedt et al., 2007; Loi et al., 2007). Each dataset selected for this study was based on Affymetrix U133A, and all of the datasets contained clinical outcome data. The raw expression data were normalized by Robust multi-array average (RMA) (Irizarry et al., 2003) and compiled. The batch effect was removed by using Bayesian Factor Regression Modeling (BFRM) (Carvalho et al., 2008). Following this procedure, a normalized gene expression dataset compiling 1,079 breast cancer samples was generated.

### Cell Culture

Human breast cancer cell lines were obtained from American Type Culture Collection (ATCC, USA). SKBR3 and BT474 cells were cultured in DMEM/F12 (GIBCO/Invitrogen, Carlsbad, CA) supplemented with 10% fetal bovine serum (FBS). All cells were maintained at 37°C in an incubator containing 5% CO_2_. Primary mouse embryonic fibroblasts (MEFs) were derived from *Ptpro* ^*+/+*^ and *Ptpro* ^*−/−*^ mice. In brief, uteri firstly isolated from 13.5-days-pregnant mice, then washed uteri with phosphate-buffered saline (PBS) and removed the head and visceral tissues. After washed with fresh PBS, the remaining bodies were minced using a pair of scissors, transferred into a 0.25% trypsin/EDTA solution (1 mL per embryo) at 37°C for 20 min. The trypsin was inactivated by DMEM with 10% FBS. The cells were cultured for 24 h at 37°C. Adherent cells were used as MEF cells.

### Plasmid Constructs and Transfection

The full-length sequence encoding PTPRO was inserted into a pCR3.1 expression vector. Lentiviral pGIPZ shRNA vectors targeting human PTPRO (pGIPZ-shPTPRO, V2LHS_226,171) and non-targeting pGIPZ control vector (pGIPZ-shCtrl) were obtained from Open Biosystems (Huntsville, AL, United States). Transfection of plasmids was performed using Lipofectamine 3,000 (Cat. No. L3000015, Thermo Fisher Scientific) according to the manufacturer’s instructions. To overexpress mouse *Erbb2* in *Ptpro* ^*+/+*^ MEFs and *Ptpro* ^*−/−*^ MEFs, the plasmid FUGW-Erbb2 (a generous gift from Prof. Yi Li, Baylor College of Medicine) was transfected into the cells, which expresses mouse Erbb2 under control of a ubiquitin promoter, using the Calcium Phosphate Transfection Kit (Invitrogen) based on the manufacturer’s instructions.

### Cell Viability Assay and Colony Formation Assay

The cell viability was examined by MTT assay. MTT and colony formation assays were performed as described previously (Feng et al., 2014; Gan et al., 2016). For MTT assay, cells were inoculated in a 96-well plate and incubated with 10-fold serial dilutions of lapatinib ranging from 0.001 to 10 μM for 72 h at 37°C. The optical density (OD) of each well was measured with a microplate reader set at 570 nm. Cell inhibition ratio (%) = [OD_control_-OD_lapatinib treatment_]/OD_control_ ×100%.

For colony formation assay, cells were trypsinized and 1,000 viable cells (depending on the experiment) were inoculated into six-well plates (in triplicate). Cells were allowed to adhere and colonize for 2 weeks. To visualize colonies, media was removed and cells were fixed in 100% methanol for 15 min and stained with crystal violet (0.1%, Sigma) for 20 min.

### Cell Death Assay

The cell death was confirmed by a photometric enzyme-immunoassay (Cat. No. 11774425001, cell death detection ELISA^PLUS^ kit, Roche Diagnostics, Mannheim, Germany) according to manufacturer’s protocol as previously described (Chandrasekar et al., 2008). In brief, the cells (1×10^5^ cells/mL) were placed in a streptavidin-coated microtiter plate with 10-fold serial dilutions of lapatinib ranging from 0.001 to 10 μM for 72 h at 37°C. After that, cells were incubated with a mixture of biotin-labeled monoclonal histone antibody and peroxidase-conjugated monoclonal DNA antibody at 37°C for 2 h. The amount of mono- and oligonucleosomes was measured as the absorbance at 405 nm with a microplate spectrophotometer (Bio-Rad, United States) after washing to remove unbound antibodies.

### Reverse Transcription-Quantitative PCR and Western Blot

Total RNA was extracted from cells using TRIzol (Invitrogen). Four-μg RNA was reverse transcribed using M-MLV Reverse Transcriptase (Invitrogen) with oligo-(dT)20 primer (Invitrogen) according to the manufacturer’s instructions. Then equal amounts of cDNA were amplified using SYBR Green PCR amplification kit (Invitrogen) with the Applied Biosystems 7,500 Real-Time PCR system (Applied Biosystems, Foster City, CA, United States), as described previously (Zhang et al., 2013; Lin et al., 2019). The results were normalized to GAPDH expression. All reactions were run in triplicate. Western blot was performed as described previously (Du et al., 2019; Xiong et al., 2020; Wang et al., 2021b). Whole-cell extracts from breast cancer cell lines. The following antibodies were used: anti-PTPRO (Cat. No. 12161-1-AP; Proteintech Group Inc., Chicago, IL, United States); anti-β-actin (Cat. No. #4967, Cell Signaling Technology, Beverly, MA, United States). Specially, the membranes of PTPRO proteins were incubated with horseradish enzyme-labeled secondary antibody for 1 h at room temperature. An enhanced chemiluminescence (IBright FL1000, Life Technologies, United States) detection system was applied to detect protein blots.

### Statistical Analysis

All statistical analyses were performed using the SPSS 19.0 statistical software package (SPSS Inc., IL, United States). The differences in PTPRO expression immunoreactivity score were compared by the Mann-Whitney U test. The statistical significance of differences was evaluated with a t-test, and means, standard error, and 95% confidence intervals were calculated using GraphPad Prism 5 (GraphPad Software Inc., San Diego, CA). Correlations between PTPRO expression and clinicopathological factors were investigated using the χ^2^ test. The correlation coefficient between variables was performed using Spearman’s rank test. Survival curves were generated using the Kaplan-Meier estimates, and the statistical significance of differences between curves was evaluated by the log-rank test. Furthermore, hazard ratios (HR) and 95% confidence intervals (CI), which were computed from univariate and multivariable Cox proportional hazards regression models (approximate proportionality was verified by visual examination of the Kaplan–Meier estimates), were used to assess associations between relapse-free survival and clinicopathological characteristics. A *p*-value of less than 0.05 was considered to be significant, and all tests were 2-sided.

## Results

### Low Levels of PTPRO Correlate With Tumor Progression and Poor Prognosis in Patients With ERBB2-Positive Breast Cancer

**FIGURE 1:**
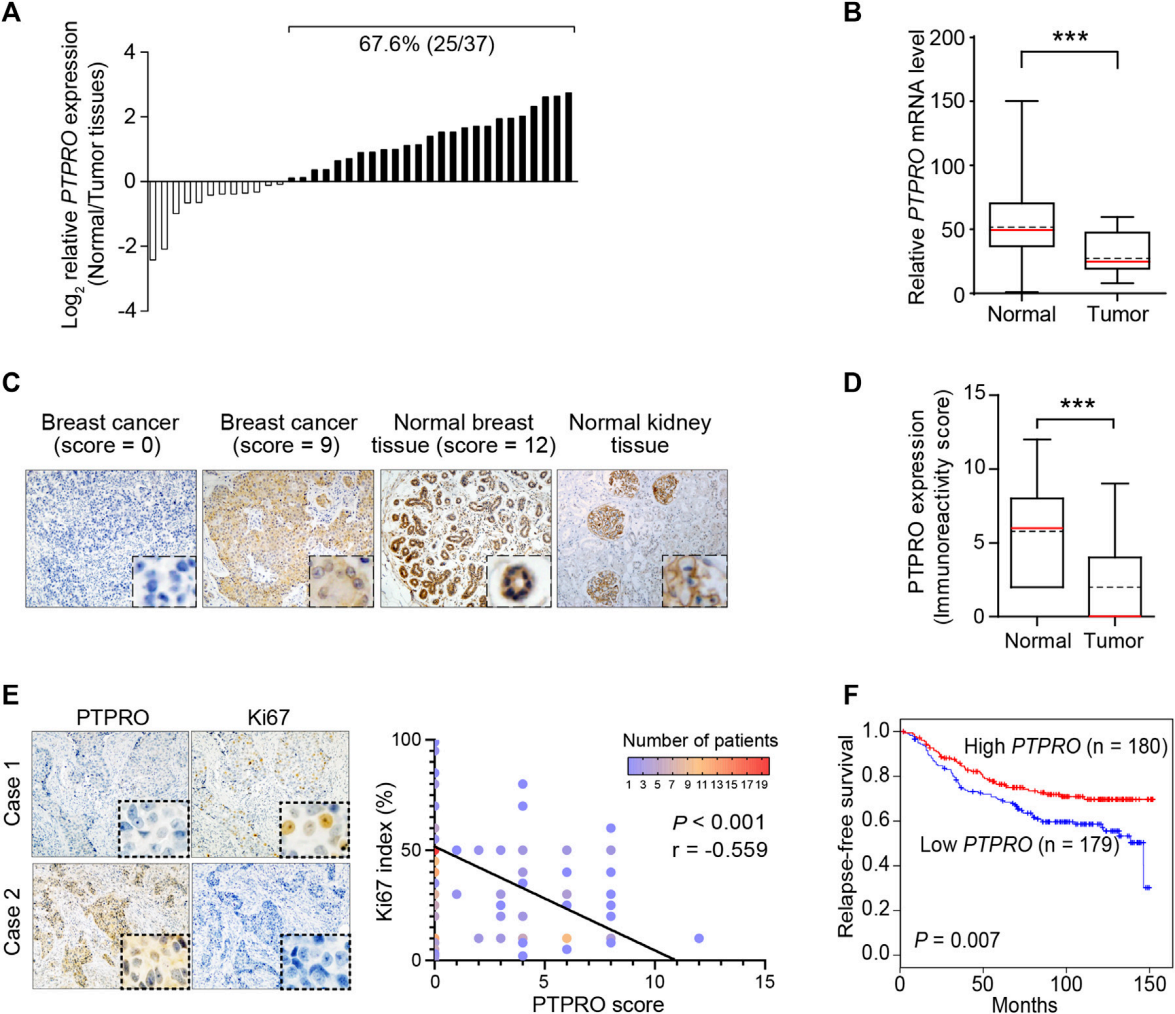
Downregulation of PTPRO correlates with poor prognosis in ERBB2-positive breast cancer patients. **(A)** The mRNA level of *PTPRO* in cancer tissues and corresponding adjacent normal breast tissues was determined in 37 breast cancer patients. **(B)** Paired t-test revealed the significant alteration of *PTPRO* mRNA in tissue samples (***p < 0.001). **(C)** Representative images are shown as IHC scores (0–9); Normal kidney and breast tissues were positive references. **(D)** The immunoreactivity scores of PTPRO in normal breast tissues and breast cancer tissues were compared using the Mann-Whitney U test (***p < 0.001). **(E)** Images of IHC staining of PTPRO and Ki67 in serial sections of two representative human breast cancer specimens (left panel); Pearson’s correlation analysis of Ki67 index and PTPRO score in 180 human breast cancer specimens (right panel). **(F)** Relapse-free survival analysis in breast cancer patients according to *PTPRO* high and low expression levels (log-rank test). These data came from the publicly available cohorts (GSE3494, GSE7390, GSE6532, GSE1456, GSE2034; the top tertile (359 cases) of *ERBB2* mRNA expression was assumed to be ERBB2-positive tumors).

To determine if PTPRO plays any role in breast cancer, we first conducted reverse transcription-quantitative PCR (RT-qPCR) to estimate the mRNA levels of *PTPRO* in 37 breast tumor tissues and found that compared with their adjacent normal tissues, the mRNA level of *PTPRO* is significantly lower in 67.6% of cancer tissues (Figures 1A,B). We then conducted IHC analysis of PTPRO on a tissue microarray (TMA) containing 180 human breast cancer samples and 18 normal breast tissues. Figure 1C showed the representative photomicrographs with PTPRO IHC scores of 0, 9, and 12 along with normal human kidneys which expresses a high level of PTPRO as a positive reference (Dong et al., 2017a). Compared with that of the normal breast tissues, PTPRO IHC scores in breast cancer samples are significantly lower (*p* < 0.001) (Figure 1D). The association between the levels of PTPRO and the clinicopathological parameters in the cohort of 180 breast cancer specimens used in the TMA was also examined (Table 1). The levels of PTPRO in poorly and moderately differentiated cancer specimens were significantly lower than that of well-differentiated cancer (*p* = 0.031). In addition, the levels of PTPRO were consistently lower in larger (>2cm, *p* = 0.013), more invasive (T3+T4 *vs*. T1+T2, *p* = 0.003), and ERBB2-positive tumors (*p* = 0.027). Of note, the levels of PTPRO were inversely correlated with the levels of Ki67 (*p* < 0.001) and the representative cases shown in Figure 1E (Left panel). This finding is consistent with the relationship between PTPRO expression and the Ki67 index of the breast cancer specimens used in the TMA (*r* = - 0.559; *p* < 0.001) (Figure 1E, right panel). These data altogether suggest that reduced PTPRO expression is associated with a wide spectrum of clinicopathological parameters in breast cancer.

Since PTPRO expression is closely related to ERBB2 status (Dong et al., 2017a), we thus further investigated the relationship between PTPRO expression and clinical outcomes in ERBB2-stratified breast cancer patients in a large dataset. In patients with lower levels of *PTPRO*, the relapse-free survival is significantly shorter than those with higher levels of *PTPRO* (*p* = 0.007) (Figure 1F). Multivariate analyses suggest that *PTPRO* can serve as an independent prognostic factor for patients with high levels of ERBB2 (HR = 0.512, 95% CI = 0.324 to 0.807, *p* = 0.004) (Table 2).

**TABLE 2:** Univariate and multivariate Cox proportional hazards model predicting survival in ERBB2-positive breast cancer patients.

Variables	Univariate analysis	*P*	Multivariate analysis	*P*
	HR (95% CI)		HR (95% CI)	
Age				
≤50 *vs.* > 50	0.752 (0.479–1.182)	0.217	0.785 (0.459–1.342)	0.376
Tumor depth				
T_3_/T_4_ *vs*. T_1_/T_2_	1.710 (1.028–2.845)	0.039	1.735 (1.045–2.881)	0.033
Lymph node metastasis				
Positive *vs.* Negative	2.079 (1.249–3.459)	0.005	1.506 (1.195–3.138)	0.007
Histologic differentiation				
Poor/moderate *vs.* Well	2.192 (1.311–3.665)	0.003	2.301 (1.394–3.798)	0.001
*PTPRO* expression				
High *vs.* Low	0.523 (0.330–0.828)	0.006	0.512 (0.324–0.807)	0.004

HR, hazard ratio; CI, confidence interval.

### Downregulated PTPRO Correlates to Lapatinib Resistance

**FIGURE 2:**
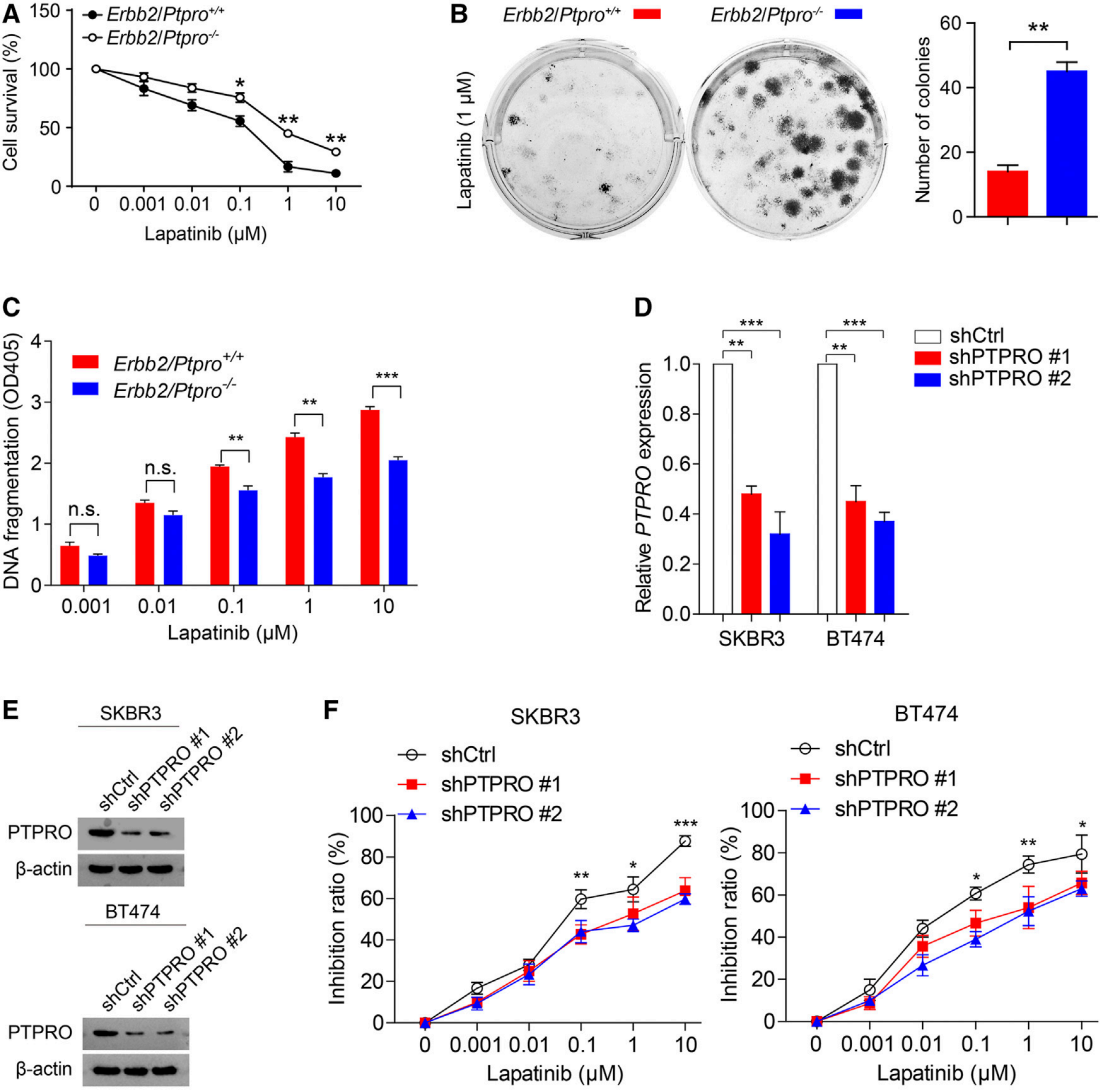
PTPRO deficiency reduces lapatinib sensitivity in *Ptpro* ^*−/−*^ and littermate-control MEFs stably transfected with *Erbb2* and ERBB2-positive breast cancer cells. **(A)** *Erbb2*/*Ptpro* ^*+/+*^ MEFs and *Erbb2*/*Ptpro* ^*−/−*^ MEFs were plated in 96-well plates and treated with 10-fold serial dilutions of lapatinib ranging from 0.001 to 10 μM for 72 h. The percentage cell viability was evaluated by MTT assay. **(B)** *Erbb2/Ptpro* ^*+/+*^ MEFs and *Erbb2/Ptpro* ^*−/−*^ MEFs were incubated with lapatinib (1 μM) for 2 weeks to allow colony formation (left panel) and the number of colonies was compared (right panel; **p < 0.01). **(C)** *Erbb2/Ptpro* ^*+/+*^ MEFs and *Erbb2/Ptpro* ^*−/−*^ MEFs were incubated with lapatinib ranging from 0.001 to 10 μM for 72 h. Cell death was determined using a Cell Death detection ELISA and absorbance was measured at 405 nm. **(D)** RT-qPCR of *PTPRO* in SKBR3 and BT474 cells with PTPRO depleted. **(E)** The protein level of PTPRO in SKBR3 and BT474 cells with PTPRO knockdown were analyzed by western blot. **(F)** Cell inhibition ratio analysis of SKBR3 and BT474 cells with PTPRO depleted was evaluated by MTT assay, 72 h after treatment with 10-fold serial dilutions of lapatinib ranging from 0.001 to 10 μM. Data were shown as the means of three independent experiments or representative data. Error bars indicate SEM; n.s., not statistically significant; *p < 0.05, **p < 0.01, ***p < 0.001 by Student’s t-test or a one-way ANOVA with post hoc intergroup comparisons, where appropriate.

**FIGURE 3:**
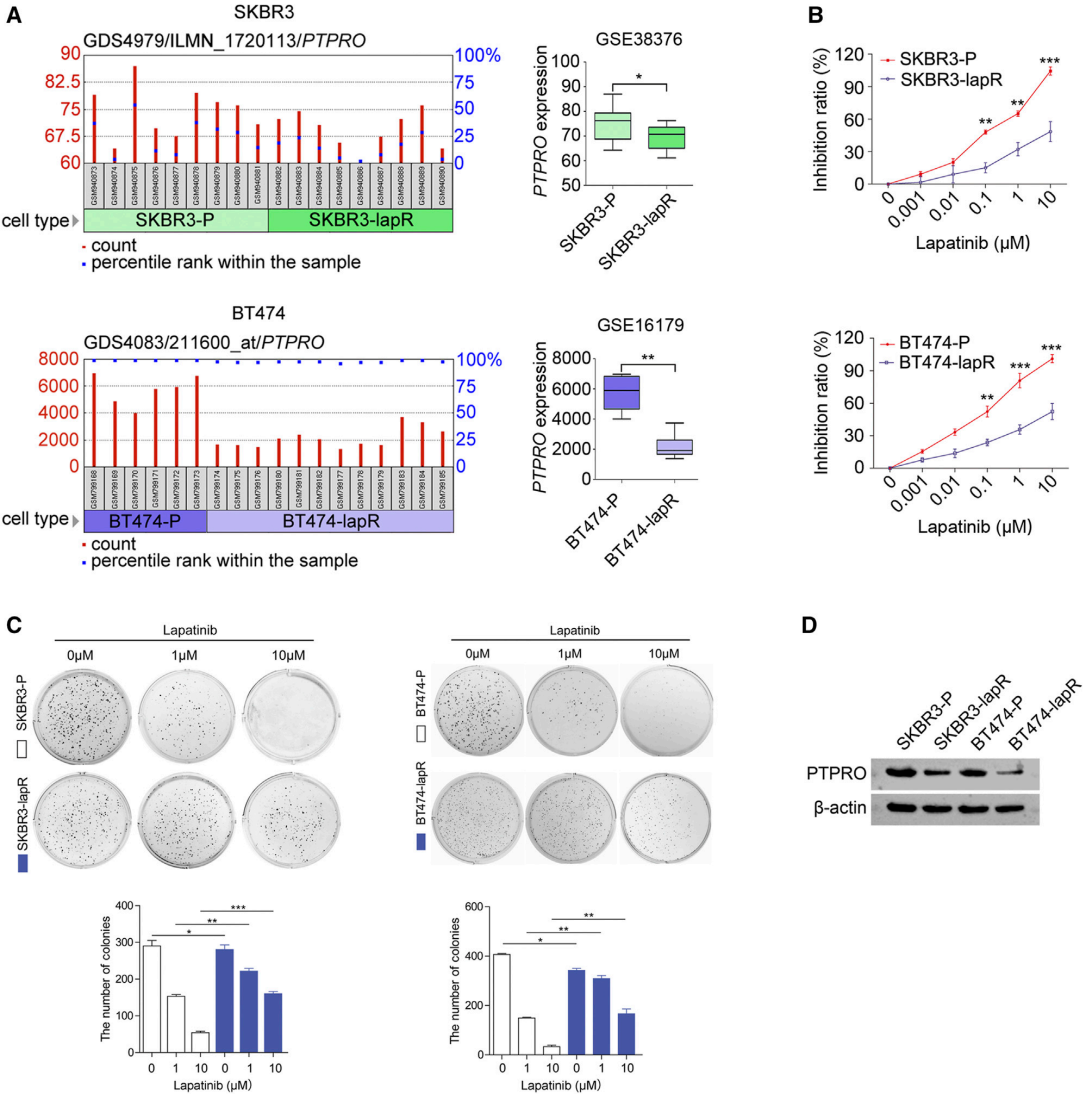
PTPRO is downregulated in lapatinib-resistant ERBB2-positive breast cancer cells. **(A)** The mRNA level of *PTPRO* in the lapatinib resistant ERBB2-positive breast cancer cells, SKBR3-lapR and BT474-lapR, compared with their parental cells, SKBR3-P and BT474-P. The dataset GSE38376 and GSE16179 were get from GEO (https://www.ncbi.nlm.nih.gov/geo/). **(B)** Inhibition ratio analysis of SKBR3-lapR and SKBR3-P (upper panel), BT474-lapR and BT474-P (bottom panel) was evaluated by MTT assay after treatment with lapatinib. **(C)** Colony formation abilities of SKBR3-lapR and SKBR3-P (left panel), BT474-lapR and BT474-P (right panel) were evaluated after treatment with lapatinib, and the number of colonies was counted (bottom panel). **(D)** The PTPRO protein expression derived from lapatinib resistant ERBB2-positive cells and their parental cells were assessed by western blot analysis. Data were shown as the means of three independent experiments or representative data. Error bars indicate SEM. *p < 0.05, **p < 0.01, ***p < 0.001 by Student’s t-test or one-way ANOVA with post hoc intergroup comparisons, where appropriate.

To investigate whether the levels of PTPRO affect lapatinib sensitivity, we generated mouse MEFs derived from two genotypic mice, *Ptpro* ^*+/+*^ *and Ptpro* ^*−/−*^, and stably expressed ERBB2 by transfecting the cells with the plasmid FUGW-*Erbb2*. When the cells were treated with either 1 μM or 10 μM lapatinib, the survival rate of *Erbb2*/*Ptpro* ^*−/−*^ MEFs is significantly higher than that of *Erbb2*/*Ptpro* ^*+/+*^ MEFs (*p* < 0.01; Figure 2A). Colony formation assay also showed that more colonies were formed by *Erbb2*/*Ptpro* ^*−/−*^ MEFs than *Erbb2*/*Ptpro* ^*+/+*^ MEFs in the presence of 1 μM lapatinib (*p* < 0.01; Figure 2B). Cell death assay suggested that *Erbb2*/*Ptpro* ^*−/−*^ MEFs can attenuate apoptotic cell death than *Erbb2*/*Ptpro* ^*+/+*^ MEFs in the presence of 0.1–10 μM lapatinib (*p* < 0.01; Figure 2C). Furthermore, depletion of PTPRO from ERBB2-positive breast cancer cells SKBR3 and BT474 also reduced their lapatinib sensitivity (Figures 2D–F). These findings altogether suggest the importance of PTPRO in lapatinib sensitivity. Next, we analyzed two public available datasets GSE38376 (Komurov et al., 2012) and GSE16179 (Liu et al., 2009) and found that the *PTPRO* mRNA level is lower in lapatinib-resistant breast cancer cells (SKBR3-lapR and BT474-lapR) than that of their parental cells (SKBR3-P and BT474-P) (Figure 3A). We then established two lapatinib-resistant ERBB2-positive breast cancer cell lines (SKBR3-lapR and BT474-lapR) in our lab following the published protocol (Bianco et al., 2008). As expected, the SKBR3-lapR and BT474-lapR cells showed their resistance to the lapatinib treatment (Figures 3B,C). More importantly, we found that the levels of PTPRO in SKBR3-lapR and BT474-lapR are significantly lower than that of their parental cells (Figure 3D). These findings demonstrated that downregulated PTPRO is correlated with lapatinib resistance.

### Overexpressed PTPRO is Capable of Reversing Lapatinib-Resistance in ERBB2-Positive Breast Cancer Cells

**FIGURE 4:**
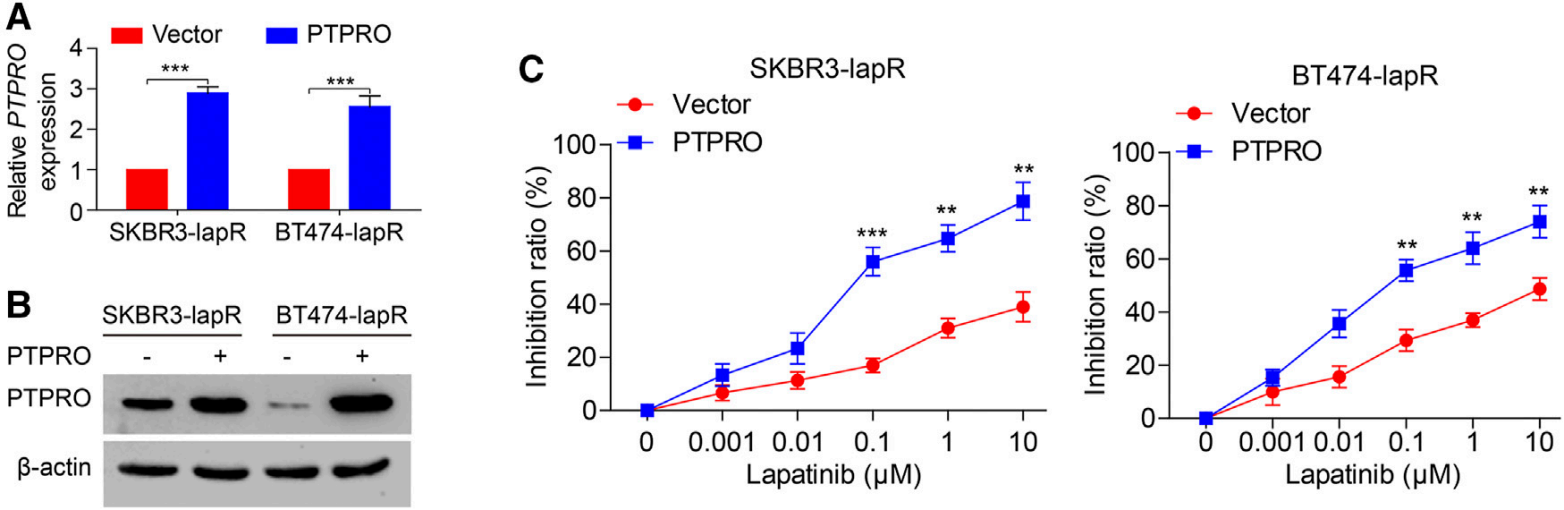
PTPRO sensitizes lapatinib in ERBB2-positive breast cancer cells. **(A)** The level of *PTPRO* mRNA in SKBR3-lapR and BT474-lapR cells with PTPRO overexpression were analyzed by RT-qPCR. **(B)** The protein level of PTPRO in SKBR3-lapR and BT474-lapR cells with PTPRO overexpression were analyzed by Western blot. **(C)** Cell inhibition ratio analysis of SKBR3-lapR and BT474-lapR cells with PTPRO overexpression was evaluated by MTT assay, 72 h after treatment with 10-fold serial dilutions of lapatinib ranging from 0.001 to 10 μM. Data were shown as the means of three independent experiments or representative data. Error bars indicate SEM. **p < 0.01, ***p < 0.001 by one-way ANOVA with post hoc intergroup comparisons, where appropriate.

We next assessed the impact of PTPRO on the lapatinib resistance in ERBB2-positive breast cancer cells. Two established lapatinib resistant ERBB2-positive breast cancer cells, SKBR3-lapR and BT474-lapR, were transfected with the plasmid overexpressing PTPRO. The mRNA and protein levels of PTPRO were then estimated by RT-qPCR and Western blot (Figures 4A,B respectively). As shown in Figure 4C, cells were treated with 10-fold serial dilutions of lapatinib from 0.001 to 10 μM for 72 h, and the inhibition ratio exhibited a dose-dependent increase in the dose range of 0.1–10 μM. Of note, both SKBR3-lapR and BT474-lapR cells become more sensitive to lapatinib when PTPRO is overexpressed (Figure 4C). Altogether, our data strongly suggest that upregulated PTPRO in ERBB2-positive breast cancer cells can enhance lapatinib sensitivity.

## Discussion

We found in this study that the expression level of PTPRO in breast cancer tissues is lower than that of the normal tissue. Further, the reduced expression level of PTPRO is associated with tumor size, poor histological differentiation, increased tumor depth, ERBB2, and Ki67 expression as well as shorter patient survival. It can also serve as an independent prognostic indicator for survival. In addition, PTPRO knockdown robustly reduced the sensitivity of the cancer cells to lapatinib. More importantly, overexpressed PTPRO is capable of reversing lapatinib resistance suggesting that upregulating PTPRO may be an effective strategy for overcoming lapatinib resistance.

The receptor tyrosine kinase ERBB2 is overexpressed in 15–20% of breast tumors and increased ERBB2 is capable of enhancing cancer cell proliferation and invasion, which is associated with a poor prognosis for overall survival (Murthy et al., 2016; Ethier et al., 2021). Therefore, targeting ERBB2 has been a strategy for drug development. ERBB2-targeted therapy drugs approved by the Food and Drug Administration (FDA), including the monoclonal antibodies trastuzumab and pertuzumab, the small molecule ERBB1/2 tyrosine kinase inhibitor (TKI) lapatinib, neratinib, tucatinib and the antibody-drug conjugate trastuzumab emtansine (TDM1), have significantly improved outcomes for patients with ERBB2-positive breast cancer (Xu et al., 2017). Lapatinib, an oral tyrosine kinase inhibitor that potently and specifically inhibits ERBB2, is now indeed a third-line therapy for ERBB2-positive disease (Vogel et al., 2010). However, lapatinib has been shown to improve progression-free survival (PFS) in patients with advanced/metastatic ERBB2-positive breast cancer (Geyer et al., 2006). Moreover, lapatinib is also approved for patients with ERBB2-positive advanced-stage breast cancer, and can show synergistic activity when used in combination with anti-ERBB2 antibodies such as trastuzumab (Guarneri et al., 2008; Medina and Goodin, 2008). A preclinical study shows that lapatinib inhibits the growth of trastuzumab-resistant ERBB2-positive breast cancer cells and increases apoptosis of anti-ERBB2 antibodies (O'Donovan et al., 2011). Evidences above suggest that lapatinib alone may be effective in treating trastuzumab-resistant ERBB2-positive patients (Blackwell et al., 2010). In addition, lapatinib is able to diffuse in the central nervous system (CNS) (Saleem et al., 2015), so it has the potential to improve the control of CNS disease compared to other monoclonal antibodies (Gril et al., 2008). Randomized trial has shown that lapatinib combined with chemotherapy reduces CNS involvement (Cetin et al., 2012). Of note, lapatinib appears to have a lower incidence of cardiotoxicity than trastuzumab, according to a comprehensive analysis of the clinical trials in metastatic breast cancer (MBC) treatment (Perez et al., 2008). Although lapatinib has many advantages such as we mentioned above, acquired resistance to lapatinib remains a major concern for its clinical utilization and lapatinib shares some of the mechanisms of resistance described for anti-ERBB2 targeted therapies, thus highlighting the importance of finding mechanisms of primary/acquired resistance.

As a member of the PTP family, PTPRO can serve as a tumor suppressor (Motiwala et al., 2003; Motiwala et al., 2004; You et al., 2012; Huang et al., 2013; Dong et al., 2017a). We have reported previously that *PTPRO* promoter hypermethylation is associated with poor survival for patients with ERBB2-positive breast cancer (Huang et al., 2013). In the current study, we showed that low expression of PTPRO is associated with poor prognosis and PTPRO could serve as an independent prognostic factor for relapse-free survival for patients with ERBB2-enriched breast cancer. Similar results have been reported in lung squamous cell carcinoma (Ming and Sun, 2017) and colorectal cancer (Asbagh et al., 2014).

Lapatinib resistance involves alterations of multiple signaling pathways including compensatory activation of RTKs, non-receptor tyrosine kinases (NRTKs), as well as the ERBB2-regulated downstream pathways (Shi et al., 2016). In addition, cross-talks among these pathways further exacerbated therapeutic resistance which makes current strategies preventing lapatinib resistance by targeting individual pathways far from ideal (Shi et al., 2016; Xuhong et al., 2019). To overcome this dilemma, we explored the possibility of preventing lapatinib resistance by focusing our attention on PTPRO, the upstream regulator of these pathways. It is known that PTPRO can dephosphorylate EGFR at Y845 and subsequently inhibit EGFR-mediated activation of SRC (Asbagh et al., 2014). PTPRO also can inactivate ERK/AKT pathways by dephosphorylating and repressing ERBB2 (Dong et al., 2017a). Therefore, our findings provide a novel therapeutic strategy to counteract lapatinib resistance. Based on the fact that PTPRO can sensitize cancer cells to different therapies (Ramaswamy et al., 2009; Asbagh et al., 2014), we overexpressed PTPRO in ERBB2-positive cancer cells and demonstrated that the overexpressed PTPRO is capable of reversing lapatinib-insensitivity. In our previous study, we explore the potentials of reexpressing PTPRO by using 5-azacytidine in ERBB2-positive breast cancer cells (Dong et al., 2017a). After treated with 5-azacytidine, cells decreased methylation leading to reexpression of PTPRO. In addition, plenty of methods currently have been reported to overexpress genes and proteins, such as lipid nanoparticles-based mRNA delivery strategy (LNPs) (Davies et al., 2021). Thus, it is also possible to overexpress PTPRO *in vivo* using LNP-formulated *PTPRO* mRNA. We will explore this in the further. Overall, through these methods above, we can overexpress PTPRO in breast cancer and overcome the resistance in patients.

Lapatinib is indicated to has good clinical activity, and acquired resistance to lapatinib remains a major concern for its clinical utilization. The efforts to study lapatinib resistance by us and others will offer the possibility of making lapatinib become a first-line drug in the further. At the same time, lapatinib treatment based on PTPRO level also reflects the significance of tumor precision treatment. Since the drugs such as trastuzumab, TDM1, neratinib and tucatinib also target ERBB2 like lapatinib, PTPRO may also reverse trastuzumab, TDM1, neratinib or tucatinib resistance. We will continue to explore and study this issue in the future. To our knowledge, this is the first to report the role of PTPRO for the response of breast cancers toward lapatinib, and upregulated PTPRO is capable of reversing lapatinib-insensitivity. However, the clinical significance of the overexpressed PTPRO needs to be validated in larger clinical cohorts.

## Data Availability

The datasets presented in this study can be found in online repositories. The names of the repository/repositories and accession number(s) can be found in the article/Supplementary Material.
