# Supplementary material for: Tyrosine Phosphatase PTPRO Deficiency in ERBB2-Positive Breast Cancer Contributes to Poor Prognosis and Lapatinib Resistance
Source: Front Pharmacol. 2022 Apr 1;13:838171. doi: 10.3389/fphar.2022.838171 (PMC9010868; doi:10.3389/fphar.2022.838171)

# Supplementary Information

1. **Supporting western blot for Figure 2D**

**
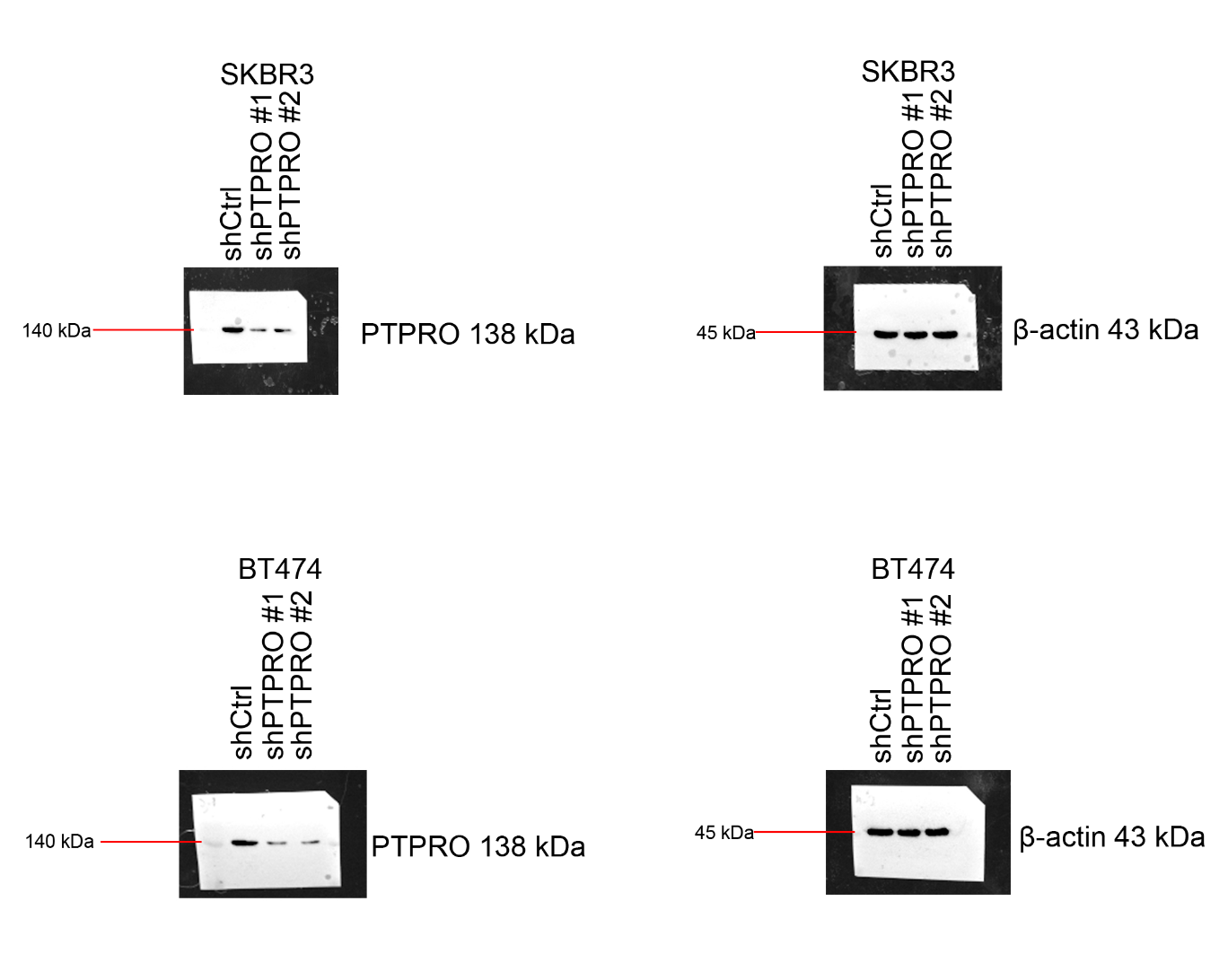
**

1. **Supporting western blot for Figure 3D**

**
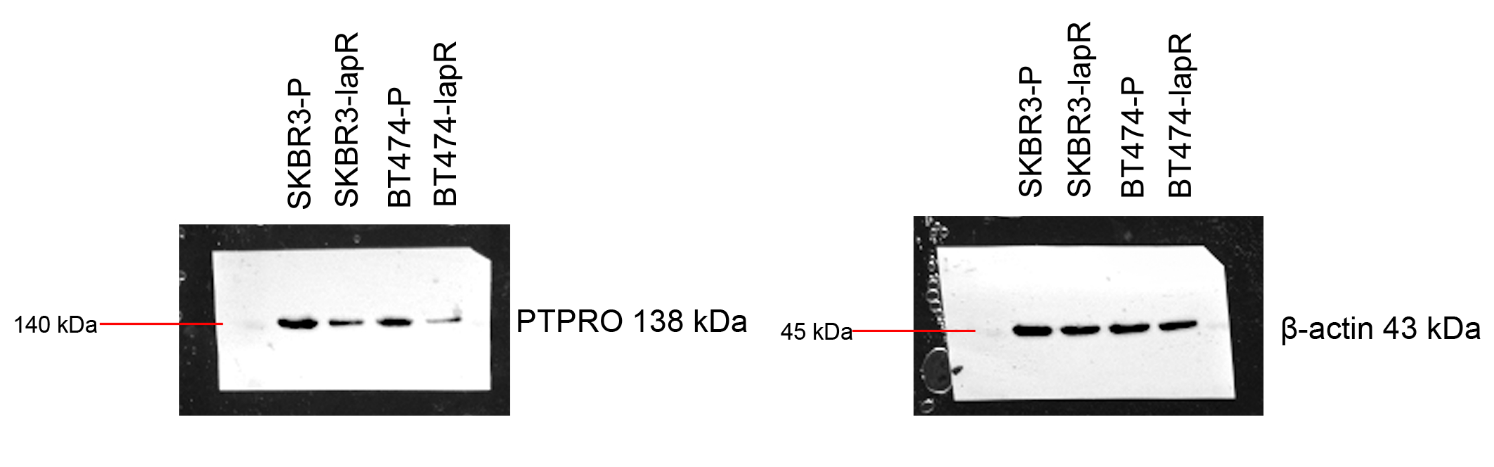
**

1. **Supporting western blot for Figure 4B**


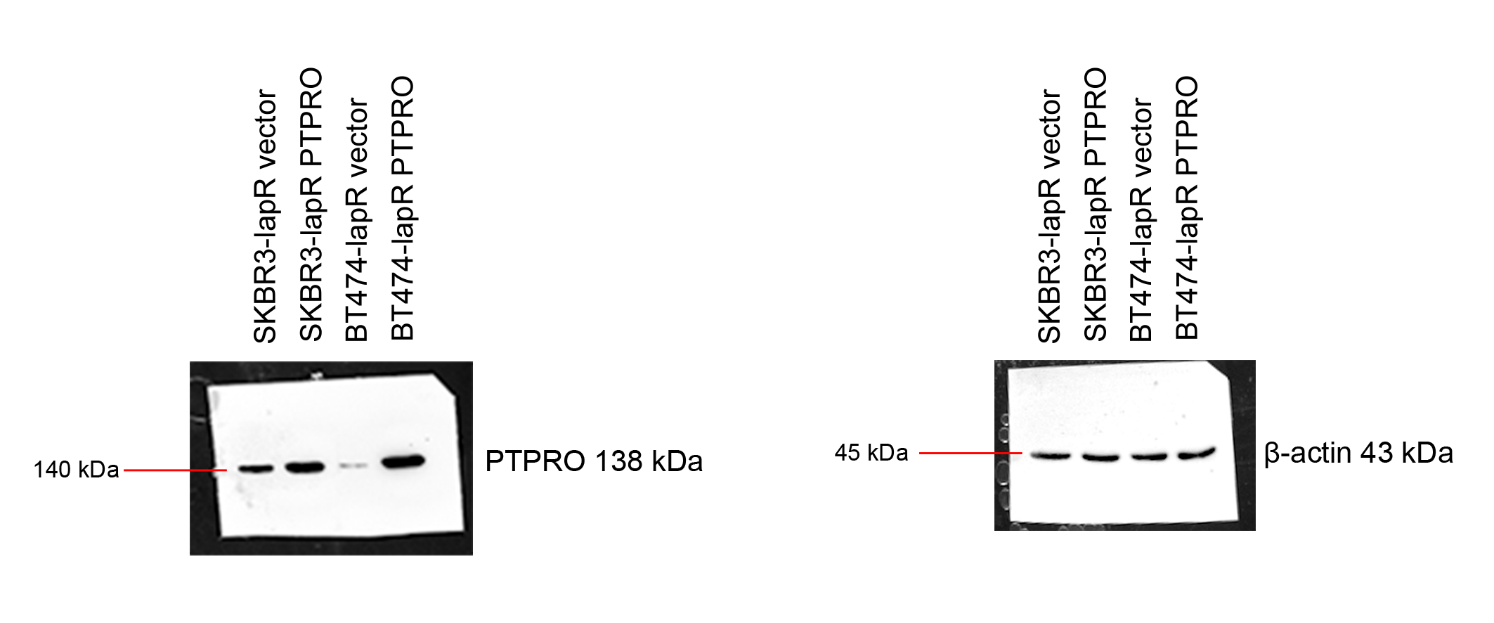

Supplement: Supplementary file 1 [file DataSheet1.DOCX]
